# A method for estimating relative changes in the synaptic density in *Drosophila* central nervous system

**DOI:** 10.1186/s12868-018-0430-3

**Published:** 2018-05-16

**Authors:** Dipti Rai, Swagata Dey, Krishanu Ray

**Affiliations:** Department of Biological Sciences, Tata Institute of Fundamental Research, Mumbai, 400005 India

**Keywords:** Squash preparation, Ventral nerve cord, Fiji^®^, Synaptic junctions, STED, *Drosophila*

## Abstract

**Background:**

Synapse density is an essential indicator of development and functioning of the central nervous system. It is estimated indirectly through the accumulation of pre and postsynaptic proteins in tissue sections. 3D reconstruction of the electron microscopic images in serial sections is one of the most definitive means of estimating the formation of active synapses in the brain. It is tedious and highly skill-dependent. Confocal imaging of whole mounts or thick sections of the brain provides a natural alternative for rapid gross estimation of the synapse density in large areas. The optical resolution and other deep-tissue imaging aberrations limit the quantitative scope of this technique.

**Results:**

Here we demonstrate a simple sample preparation method that could enhance the clarity of the confocal images of the neuropil regions of the ventral nerve cord of *Drosophila* larvae, providing a clear view of synapse distributions. We estimated the gross volume occupied by the synaptic junctions using 3D object counter plug-in of Fiji/ImageJ^®^. It gave us a proportional estimate of the number of synaptic junctions in the neuropil region. The method is corroborated by correlated super-resolution imaging analysis and through genetic perturbation of synaptogenesis in the larval brain.

**Conclusions:**

The method provides a significant improvement in the relative estimate of region-specific synapse density in the central nervous system. Also, it reduced artifacts in the super-resolution images obtained using the stimulated emission depletion microscopy technique.

**Electronic supplementary material:**

The online version of this article (10.1186/s12868-018-0430-3) contains supplementary material, which is available to authorized users.

## Background

Synapses are characterized by the presence of presynaptic active zones (AZs) and postsynaptic densities (PSDs) separated by a synaptic cleft [1]. AZs are the vesicle docking and neurotransmitter release sites associated with an electron dense cytomatrix [2–9]. The PSDs on the postsynaptic membrane is constituted by neurotransmitter receptors, channels, adhesion molecules and signaling components [1]. The bulk of synaptogenesis occurs during early development, but synapses can constantly form in the adult brain as well [10–13]. Development of behavior requires formation of a large number of new synapses and modification of existing ones, resulting in a compact organization of central nervous system (CNS). At both stages, embryonic and adult, activity-dependent refinement of synaptic connections takes place [14–19]. This dynamic process is assisted by molecular remodeling of AZs and PSDs as studied in both vertebrate and invertebrate systems [20–25]. Further, these structural changes are functionally associated with alterations in neurotransmitter release during synaptic plasticity [23, 26, 27]. However, most of these studies were carried out either at neuromuscular junctions (NMJs) or in cultured hippocampal neurons, which cannot be generalized. Molecular mechanisms underlying central synaptogenesis and synaptic plasticity during development at a global scale is still poorly understood.

A major limitation is to estimate the total synapse number in the CNS. In general, synapse estimation involves serial sectioning of brain samples and imaging under transmission electron microscope (TEM). Subsequently, a sampled estimate is established from 3D-reconstructed data of the entire synaptic field [28–32]. Synapses are counted in the serial electron microscopic reconstructions using unbiased stereological methods like optical dissector method [33, 34], and size frequency method [35]. However, sophisticated instrumentation and stringent sample preparation make it expensive and invasive. Generating and aligning these serial electron micrographs and their analysis is a complex procedure involving highly skilled labor and expensive instrumentation. Also, sample preparation is prone to generate artifacts. Even after such rigorous process, one ends up with a sample survey estimate with small sampling frequency [36]. Further, the low throughput of this technique makes it difficult for making gross comparisons amongst large volume samples.

A relatively more straightforward method is to label these synapses using fluorescent markers and obtain their optical sections using light microscopy to reconstruct the entire imaging field. The primary challenge in using conventional light microscopy is the diffraction-limited size of the active synaptic zone and high synaptic density in the CNS [37–42]. It does not allow resolution of individual synapses due to strong background signal from out of focus light. New approaches to quantify synapses and resolve their nanoscopic organization are adopted with the advent of super-resolution microscopy (SRM). Recently, few studies in *Drosophila* using the stimulated emission depletion (STED) and stochastic optical reconstruction microscopy (STORM) techniques were able to reveal the synaptic ultrastructure with relatively less tissue invasion [27, 37–44]. Despite its capacity to resolve at the nanoscale, it was most effective in resolving NMJs or in selectively marked neurons. In CNS, high tissue thickness, density of fluorescent signal, and autofluorescence reduce the signal to noise ratio. High tissue scattering and depth aberrations also introduce specific imaging artifacts. Furthermore, SRM requires fluorophores with efficient binding properties [45, 46], and expensive instrumentation.

A few studies have attempted to combine the advantages of both light and electron microscopy by applying correlative light and electron microscopy (CLEM) technique to brain tissue [40]. Moreover, automation of tissue sectioning in block-face scanning EM (BF-SEM) is a step further to create a 3D reconstruction of entire tissue to reduce time and labor requirement [47]. The disadvantage of this technique was in image analysis of the 3D reconstructed image to identify desired structures and automate their counting [36]. Most of the existing reports of quick estimation of gross synapse number in the CNS are descriptions of the redistribution of presynaptic proteins within neurons and their accumulation in cell bodies using relative intensity estimates [48–51]. For example, Dey et al. [52] calculated the overall volume occupied by the presynaptic markers in the neuropil region in ventral nerve cord of *Drosophila* to correlate the effects of altered Rab4 transport. Although it provided an indirect estimate, assuming that each synapse occupies nearly equal volume in the neuropil, it is unclear whether that would correlate to the number of synapses.

Here, we present a simple sample preparation technique useful for quick and gross assessment of synapses density in the central nervous system of *Drosophila* larvae with large area sampling. The squash preparation method preserves overall morphology and structural integrity of neurons in the ventral nerve cord of *Drosophila.* Automated morphometric analysis of confocal images of the preparations using Fiji^®^ provided an estimate of a total number of synaptic AZs marked by Bruchpilot immunostaining within each neuromere. Bruchpilot is a key component of AZs in *Drosophila* which is orthologous to human ELKS/CAST family of proteins [53, 54]. It is required for tethering vesicles and clustering of Ca^2+^ channels at the active zones [43]. It helps to establish the characteristic “T-bar” structure at the active zones [53] and has been extensively used as a bonafide marker for synapses in the NMJs as well as in the CNS of *Drosophila* [27, 38–40, 42–44, 53]. Our protocol provides a better clarity of synapses for morphometric analysis. Importantly, it offers a quick survey with large sampling at a relatively less labor investment.

## Methods

### Larval aging

*Drosophila melanogaster* adult flies were maintained in vials and bottles containing standard cornmeal media with 3:1 ratio of female to male. These flies were transferred to fresh media vials for egg laying at 25 °C. Eggs laid for the next 1 h were collected and kept at 25 °C for aging until 78 h after egg laying (h AEL).

### Dissection of the larval ventral nerve cord

Larvae aged for a designated time after egg laying (h AEL) were taken out of vials by using a wet brush. They were transferred to a petri dish with a drop of water. The cleaned up larvae were transferred with a drop of 1× Phosphate Buffered Saline (PBS, pH 7.2, 137 mM NaCl, 2.7 mM KCl, 10 mM Na_2_HPO_4_, 1.8 mM KH_2_PO_4_) onto a second petri dish containing Sylgard silicone bed (Sylgard 184 Kit from Dow-Corning Inc. USA). The central nervous system (CNS) containing optic-lobes and the VNC was dissected from the larva and transferred onto a lysine coated slide with 100 µL PEM buffer (100 mM PIPES, 2 mM EGTA, 1 mM MgSO4, pH 6.95), incubated for 2 min, and further processed as described below.

### Squash preparation of larval ventral nerve cord to resolve the synaptic contacts

CNS placed on the slide was gently covered with a 40 × 20 mm^2^ coverslip, which was allowed to settle without applying any external pressure. This procedure mildly stretched the tissue without grossly disrupting the morphology. The mount was then dipped in liquid nitrogen for 30 s, and then the coverslip was flipped using a razor blade while it was still frozen. This operation exposed neuropil by removing layers of cortical cells which were stuck onto the coverslips. The remaining tissue on the slides was fixed by incubating the slide immediately in a drop of 4% Paraformaldehyde (PFA) in PEM on ice for 10 min, which was followed by three 1-min rinses in PBS. Subsequently, the tissue was permeabilized in a drop of PBS containing 0.3% Triton-X-100 (PTX) for 20 min, blocked for 30 min in PTX containing 1 mg/ml Bovine Serum Albumin (PBTX) at room temperature. To mark the synaptic contacts, the slide was incubated with a drop of suitable antibodies diluted in PBTX for 1 h at room temperature which was followed by three 1-min rinses in PBTX, incubation in secondary antibodies diluted 1:400 in PBTX for 1 h, and a final set of 3× rinse in PBTX. The tissue samples were then mounted under a coverslip (18 × 18 mm^2^ and 0.17 mm thickness) with a drop of Vectashield^®^ (Vector Laboratories Inc., USA) for confocal imaging or Mowiol^®^ 40-88 (Sigma-Aldrich) mounting medium for super-resolution imaging. List of antibodies used is provided below in Table 1.

**Table 1:** Antibodies used to label synapse

Antibody	Source	Working dilution
Mouse monoclonal to Bruchpilot	DSHB (nc82)	1:200
Goat Anti-Mouse IgG1 Alexa Fluor 647 conjugated	Invitrogen (A-21240)	1:400
Tetramethylrhodamine α-bungarotoxin	Sigma-Aldrich (T0195)	1:200
Goat Anti-Mouse IgG-Abberior^®^ STAR 635	Sigma-Aldrich (40734)	1:400

### Image acquisition and analysis

Optical slices were acquired for each neuromere hemisegment in the A3-A6 abdominal segments of the VNC using Olympus FV1200 Laser Scanning Confocal Microscope under identical acquisition conditions using a 40 × 1.3 NA objective, 3.6× zoom and a pixel resolution of 0.17 × 0.17 µm^2^. The acquisition parameters viz., laser power, PMT gain, scan speed, optical zoom, offset, step size, pinhole diameter, etc., were kept constant for each experimental data set. For confocal and STED comparison, images were collected using 93× glycerol 1.3 NA objective on Leica SP8 TCS STED 3X. All images were deconvolved with Huygens Professional version 16.10 (Scientific Volume Imaging, The Netherlands, http://svi.nl). Images were processed in Fiji^®^ and analyzed using “3D object counter plugin” [55] of the Fiji^®^ software as described further.

### Statistics

Statistical analysis and representation of the data were carried out using OriginPro^®^ (http://www.originlab.com). All data are presented in the box and whisker plots. Box indicates the first and third quartiles. In the boxplots, the band and small square represent the median and mean, respectively. Pairwise comparisons were made using one-way ANOVA and Bonferroni’s test to test for the significance.

## Results

### Squash preparation of the ventral nerve cord did not alter the morphology of tissue and distribution of synaptic proteins

**Fig. 1:**
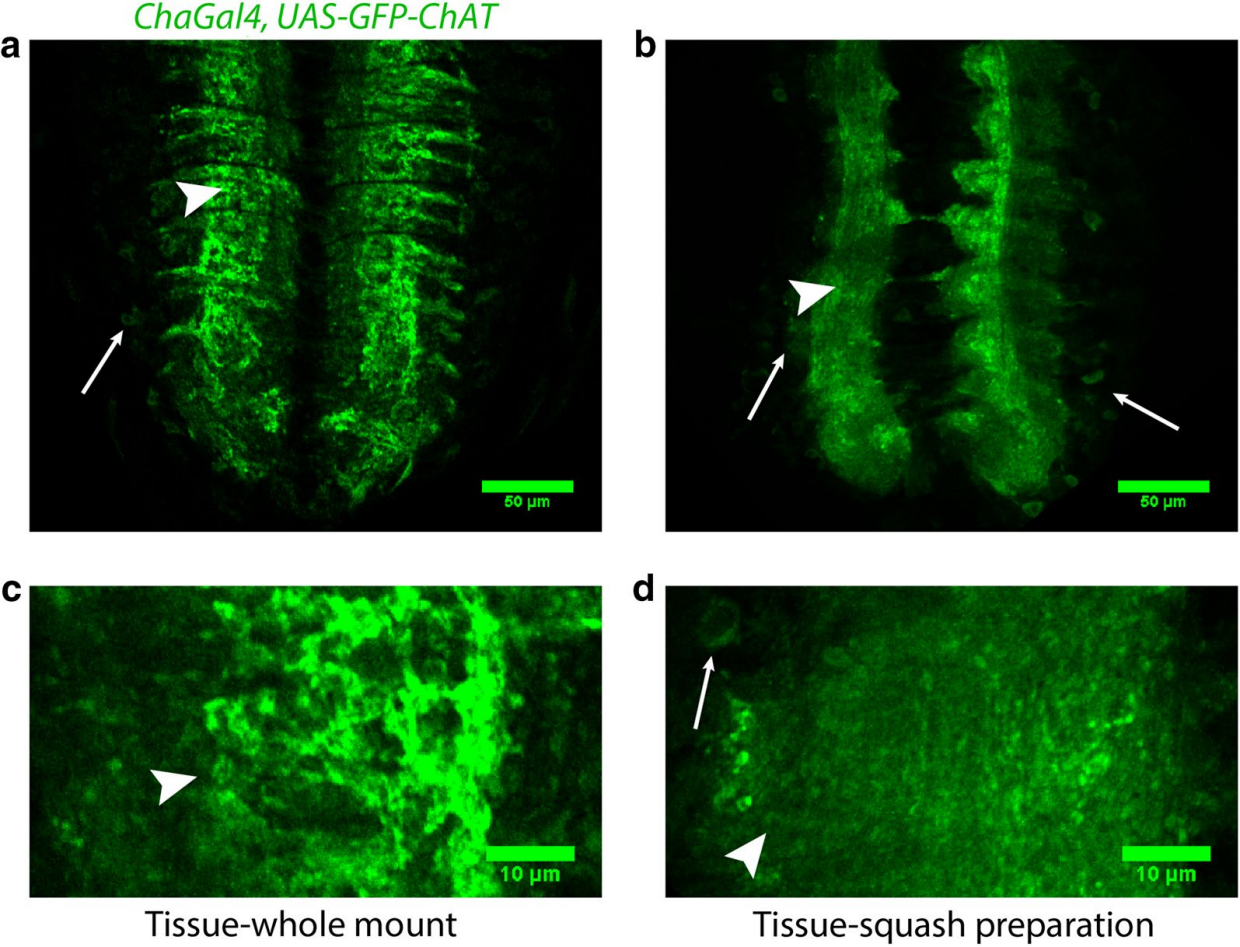
Comparison of overall morphology of VNC in whole mount and squash preparation. Confocal images of single optical sections from the VNCs of *chaGal4, UAS*-*GFP*-*ChAT* larvae obtained from whole mount (**a**, **c**) and squash (**b**, **d**) preparations. **a**, **b** Represents VNC at ×1 zoom, while (**c**, **d**) represents neuromere hemisegment at ×3.6 zoom. The overall morphology of the tissue and the neuronal connections are retained in the squash preparation. It is judged by scrutinizing the organization of commissures in the neuropil (arrowheads) and cortical region (thin arrows). Magnification ×40 oil objective, N.A. 1.3; Scale bars: **a**, **b** 50 µm, **c**, **d** 10 µm. The experiments were performed using a set of 5–10 isolated VNCs, and the images represent the majority observation amongst each set

The idea behind this method was to expand the tissue so that it would physically resolve the synaptic junctions without disturbing the tissue architecture and overall morphology. To examine the tissue morphology, we expressed *UAS*-*GFP*-*ChAT* in the cholinergic neurons using *chaGal4* marking the entire neuron. Results suggested that the tissue morphology remained intact in this procedure (Fig. 1). Moreover, the localization and distribution of the soluble presynaptic protein, Choline acetyltransferase (ChAT), were unaltered. However, the total fluorescence level seemed reduced. ChAT being a soluble protein, a small amount of the antigen might have been extracted out from the tissue during this procedure, suggesting that this method cannot be used to estimate the quantity of soluble antigen. Nonetheless, the preservation of tissue morphology allowed us to use this method to mark the synaptic contacts by labeling them using an antibody against Bruchpilot, a structural component of active zone membrane.

### Milder fixation, longer permeabilization and longer incubation with antibody gave optimum Bruchpilot staining

Next, we ascertained whether Bruchpilot antibody would label the synaptic contacts in VNCs after the squash preparations. For optimum Bruchpilot staining, we tried multiple combinations of fixation—altering the concentration of fixative, buffers and incubation times. We expressed *UAS*-*GFP*-*ChAT* in the cholinergic neurons using *chaGal4* to compare the VNC morphology between the whole mount preparation (Fig. 1a and c) and the squash preparation (Fig. 1b and d). Initially, the brain was dissected in 1× PEM buffer and then incubated it in 4% PFA solution made in 1x PEM buffer for 10, 20, and 40 min before squashing. It was followed by a 10 min post-fixation in 4% PFA in 1× PEM buffer and three washes with 1× PBS, 1 min each. Squash preparations were then incubated with the primary antibody diluted in 0.1% PBTX for 10 min at room temperature followed by three washes in PBTX, 1 min each. The same step was repeated for labeling with the secondary antibody, and then the tissues were mounted in a drop of Vectashield^®^ (Vector Laboratories Inc., USA) under an 18 × 18 mm^2^ coverslip of thickness 0.17 mm. This procedure retained the antigens but distorted the tissue morphology (Additional file 1: Figure S1B and C) and GFP-ChAT localization in neurons. The integrity of the tissue fell apart, and small gaps were observed in the tissue which increased with the pre-squash incubation time in fixative. Also, the intensity of the Bruchpilot staining was poor (Additional file 1: Figure S1E and F). In contrast, the morphology was better preserved (Additional file 1: Figure S1A) when squash was prepared using tissues without pre-fixation, although Bruchpilot staining was still poor (Additional file 1: Figure S1D).

Since prefixation before the squash preparation resulted in poor Bruchpilot staining and caused tissue distortions, we tried squash preparations without pre-fixation followed by snap freezing and postfixation for 10 min (Additional file 2: Figure S2D), 20 min (Additional file 2: Figure S2E) and 40 min (Additional file 2: Figure S2F). This time, the morphology was retained, but Bruchpilot staining was still poor and degraded further with longer pre- or post-fixation times (Additional file 2: Figure S2B, C, E, and F). To troubleshoot the problem, we tried various permeabilization treatments—0.3% PTX (Additional file 2: Figure S2G), 0.1% PTX (Additional file 2: Figure S2H) and 0.05% PTX (Additional file 2: Figure S2I) for 5 min, among which 0.3% PTX treatment permeabilized the tissue better (Additional file 2: Figure S2G). Still, it was difficult to visualize the antigen.

**Fig. 2:**
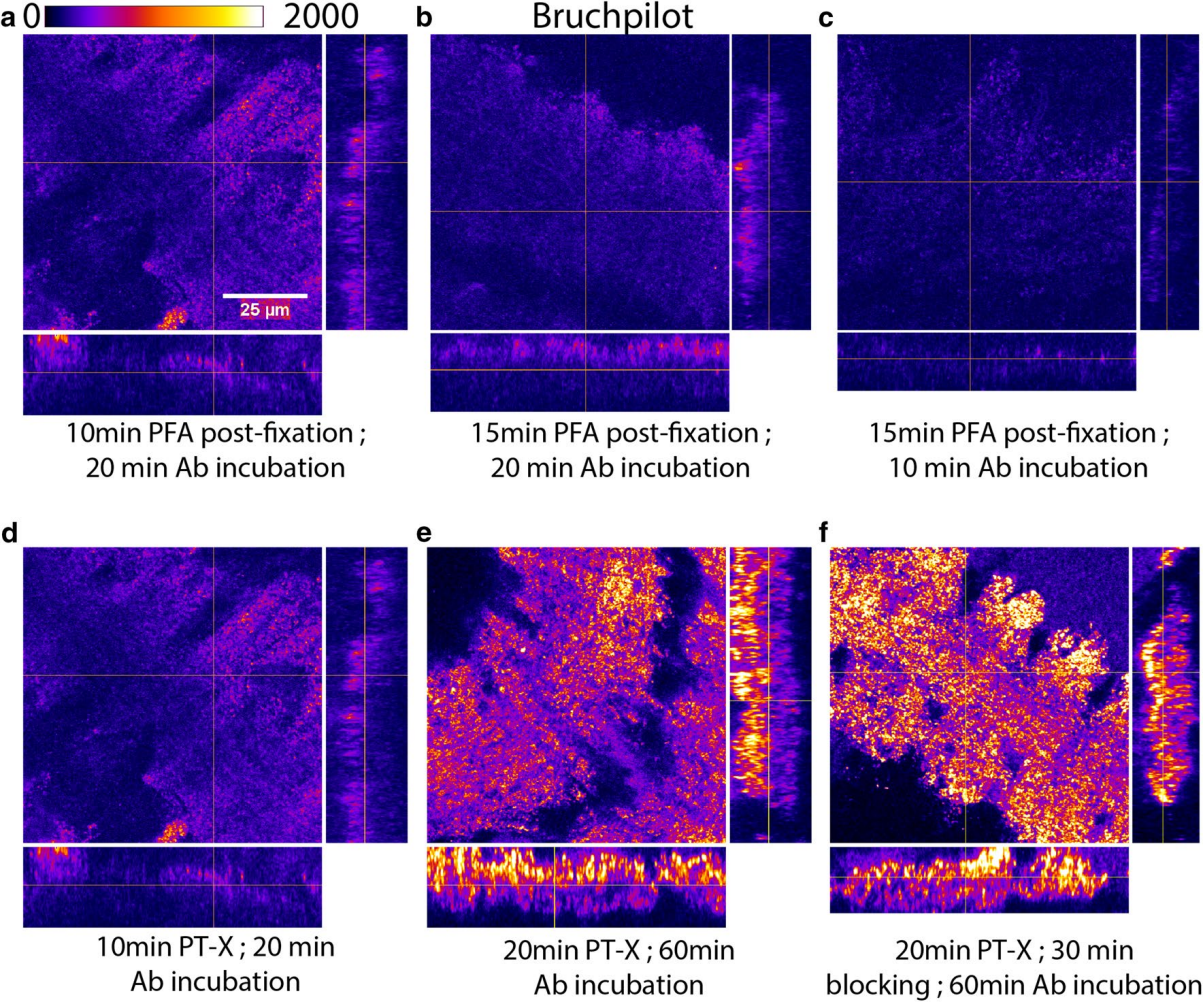
Milder fixation, longer permeabilization, and incubation with antibody gave optimum Bruchpilot staining. Squash preparation of VNC of *Drosophila* 3rd larval instar. **a**–**c** Bruchpilot staining with different combinations of incubation periods for post-fixation and antibody (Ab) incubation with 0.3% PT-X permeabilization for 10 min. **d** Milder post-fixation for 10 min with 10 min 0.3% PT-X permeabilization. **e** 20 min 0.3% PT-X permeabilization with milder post-fixation for 10 and 60 min Ab incubation. **f** 20 min 0.3% PT-X permeabilization and 30 min 0.1% PBT-X blocking with milder post-fixation for 10 and 60 min Ab incubation. Magnification ×40 oil objective, N.A. = 1.3; Scale bars: 25 µm. The experiments were performed using a set of 5–10 isolated VNCs, and the images represent the majority observation amongst each set

Next, we tried a combination of incubation periods for post-fixation and antibody incubation with 0.3% PTX permeabilization for 10 min without pre-squash fixation (Fig. 2a–c). We also increased 0.3% PT-X permeabilization up to 20 min (Fig. 2e, f) with milder post-fixation for 10 min (Fig. 2d–f).

**Fig. 3:**
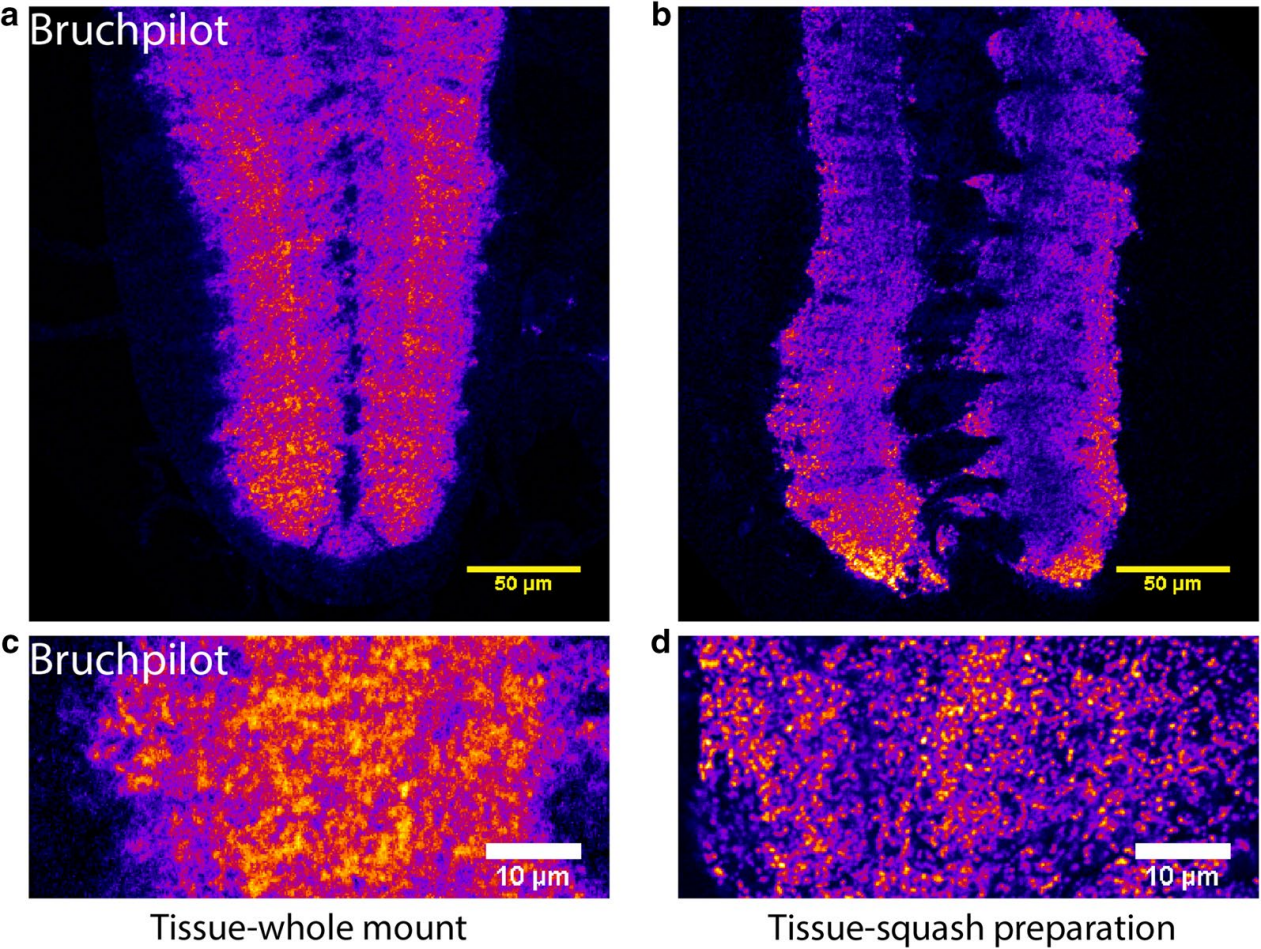
Squash preparation of VNC provided better clarity of synaptic junctions over whole mount preparation. Confocal images of single optical sections from the VNCs of third instar larvae obtained from whole mount (**a**, **c**) and squash (**b**, **d**) preparations stained with Bruchpilot antibody. **a**, **b** Represents VNC at ×1 zoom, while (**c**, **d**) represents neuromere hemisegment at ×3.6 zoom. The squash preparations revealed sharper and better clarity images of synaptic junctions (**b**, **d**) as compared to whole mount (**a**, **c**). Magnification ×40 oil objective, N.A. = 1.3; Scale bars: **a**, **b** 50 µm, **c**, **d** 10 µm. The images presented here have been consistently observed in more than 10 VNC preparations in each case

Amongst them, 10 min post-fixation with 4% PFA in PEM buffer, 20 min of permeabilization with 0.3% PTX, and 60 min of incubation with the primary and secondary antibodies produced an optimum result (Fig. 2f). This combination was finally chosen for antibody labeling of squash preparation and image acquisitions to count the synaptic contacts in larval VNC of *Drosophila.* This procedure provided a better clarity of synapses in the VNC neuropil for the assessment of synaptic junctions (Fig. 3).

### Estimation of synaptic contacts from squash preparation

Synaptic contacts were estimated from the centermost optical slice of each neuromere hemisegment, in which commissures were visible, using 3D object counter plugin of Fiji. The 3D object counter plugin of Fiji applies an auto-threshold-returned total number of contiguous voxel elements in the image field. The number of 3D objects and that of the contiguous voxels contributing to the 3D objects in an optical slice can be easily estimated using this algorithm (Additional file 3: Figure S3). For our purpose, a size filter was applied manually. It corresponds to the minimum volume of a diffraction-limited image, i.e., 0.09 μm^2^ and a maximum cut off at 6.0 μm^2^. We reasoned that the synaptic boutons, as previously estimated [27, 31, 38–40, 42], would occupy an area of 0.03–6 µm^2^ in central neuromeric slices. The confocal microscope would not allow clear resolution of the Bruchpilot punctae within a synapse or amongst several closely spaced synapses unless they are separated by 300 nm. Therefore, often multiple synapses would appear as a single 3D object in the image. Hence, the total number of such elements would vary widely depending on the squash condition. However, the total number of voxels contributed by all the 3D objects within a neuromeric volume would be consistent. Since regular optical microscopy does not resolve the synaptic junctions, this plugin only helps to estimate the gross area occupied by the synaptic contacts within a neuromere in terms of the number of voxels. We interpreted this volume as a gross estimate proportional to the number of synaptic junctions in the region. The upper limit of the single 3D object was arbitrarily chosen after observing several images.

The conjecture was further supported by the STED imaging which provided nearly 60 nm resolution. It suggested that the lower bound cut off is consistent with a single synaptic bulb composed of several Bruchpilot punctae, and upper limit included multiple adjoining synapses. However, the STED acquisition and image analysis were tedious and highly time-consuming. Therefore, the squash preparations provided a quick and reasonably refined estimate of the volume occupied by the synaptic contacts stained by the Bruchpilot in the neuropil region.

### Super-resolution microscopy revealed synaptic contacts with better clarity in squash preparations

**Fig. 4:**
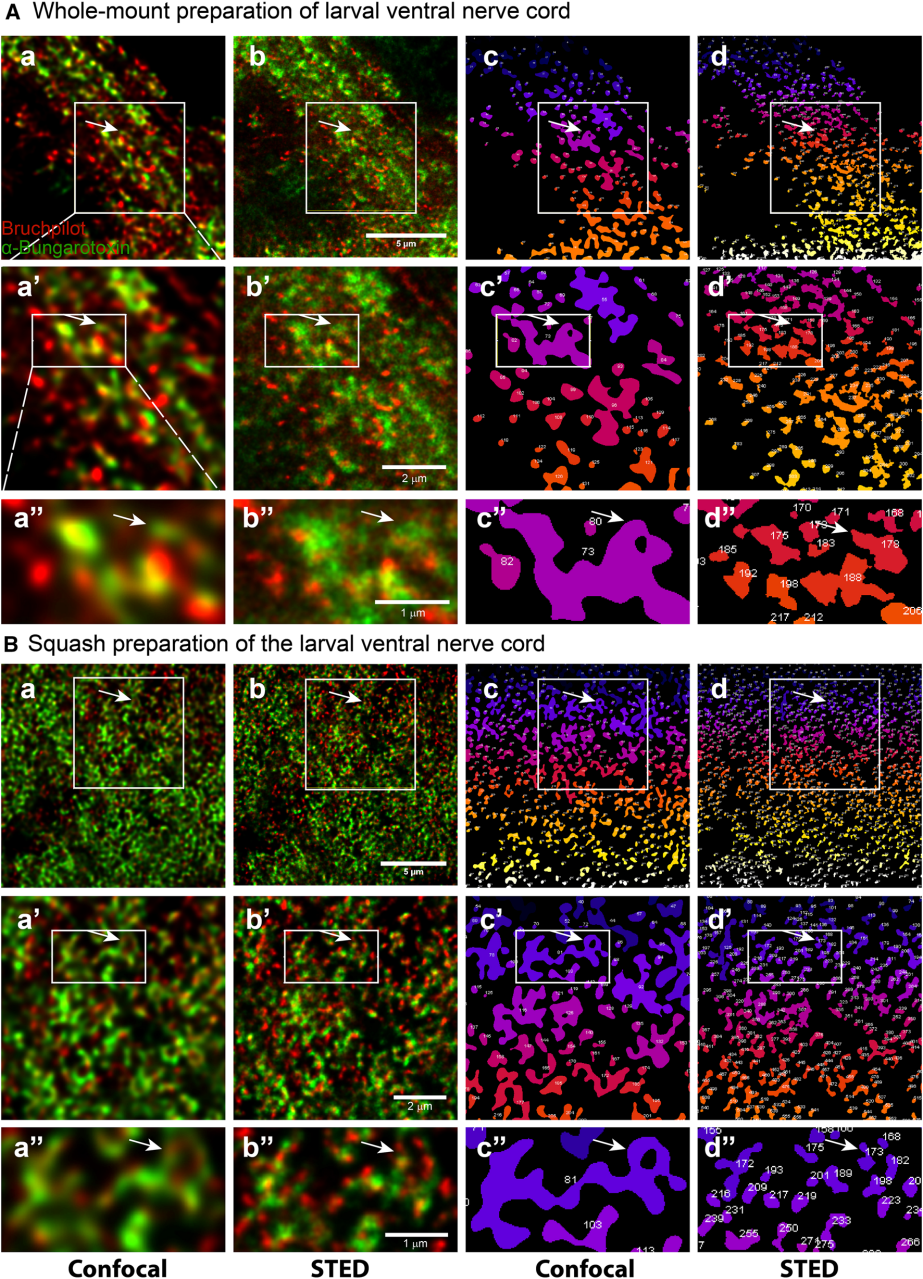
Confocal and Super-resolution imaging of Bruchpilot and Bungarotoxin staining in the whole mount and squash preparations. **A** Whole-mount preparation and **B** squash preparation of abdominal neuromere of *Drosophila* 3rd larval instar VNC showing Bruchpilot (red) and α-Bungarotoxin staining (green). The images were collected from the same specimen sequentially using confocal (a–a″), and STED (b–b″, c–c″, d–d″). The object-maps of synapses or synaptic bulbs, as shown in a–a″ and b–b″, respectively, were generated from the same regions using 3D object counter plugin of Fiji^®^. Magnification ×93 glycerol objective, N.A. = 1.3; Scale bars: 5 µm. a′, b′, c′, d′ are enlarged view of region shown in box in a, b, c, d while a″, b″, c″, d″ are enlarged view of region shown in box in a′, b′, c′, d′. Arrows in A point to a single synaptic bulb shown in whole-mount while arrows in **B** point to a single synaptic bulb shown in squash preparation. The images presented here are similar to the observations made in 3 such independent VNC preparations. In each preparation, the results are consistent amongst the A4–A6 abdominal hemisegments. All panels in a row are presented in the same scale as shown in one of the panels in each row

To understand the identity of the Bruchpilot punctae observed in the confocal images of both the whole-mount and squash-preparation specimen, we simultaneously imaged them using the Stimulated-Emission-Depletion (STED) Microscopy. The wild-type ventral nerve cord stained with Bruchpilot and tetramethylrhodamine-conjugated α-bungarotoxin revealed unresolved punctae juxtaposed with each other in the whole-mount preparations (arrows Fig. 4A-a). The STED images of the same region provided uncharacteristically distributed Bruchpilot staining associated with the bungarotoxin punctae (arrows, Fig. 4A-b). A similar region of the abdominal ventral nerve cord imaged from the squash-preparation specimen provided a much better clarity (arrows, Fig. 4B-a) with well demarcated Bruchpilot and bungarotoxin stained zones. The STED images of the same region highlighted clear Bruchpilot punctae juxtaposed to the bungarotoxin stained ones as expected (arrows, Fig. 4B-b). The spatial correlation between the Bruchpilot and bungarotoxin staining amongst the confocal and STED images were very good in the squash-preparation specimen. It was further highlighted by the 3D object counting analysis using Fiji^®^.

### Squash preparation segregates coalesced entities into smaller discretely observable units

**Fig. 5:**
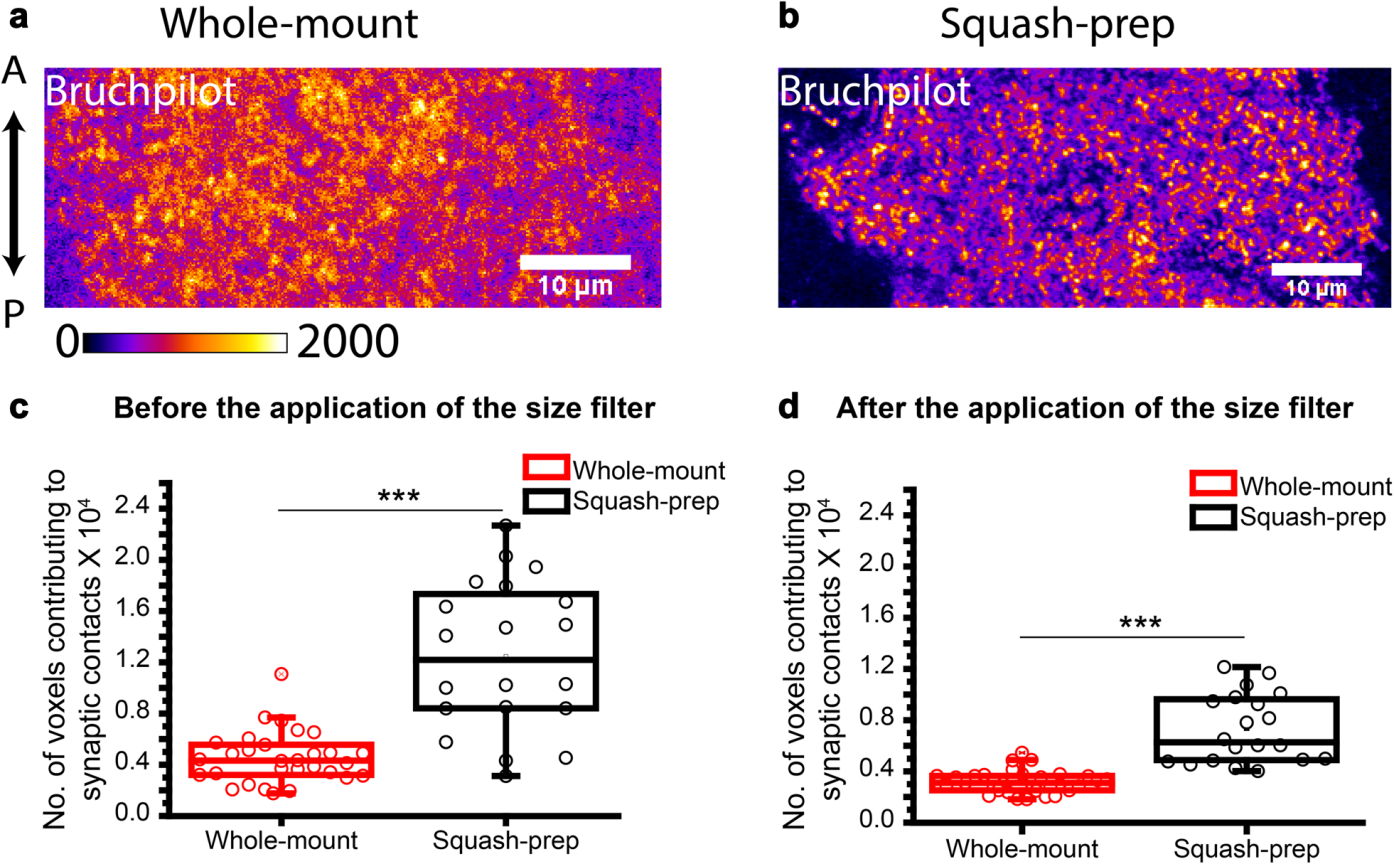
Estimation of the volume occupied by synaptic contacts in whole mount and squash preparations of VNC. **a** Whole-mount and **b** squash preparation of A6 abdominal segments of 90 h AEL VNCs from *cha*-*Gal4, UAS*-*GFP*-*ChAT* stock were stained with the Bruchpilot antibody. Images are represented in a pseudocolor scheme. Magnification ×40 oil objective, N.A. = 1.3; Scale bars: 10 µm. **c**, **d** Box plots depict quantifications of synaptic contacts before and after applying the size filter. Pairwise comparisons were made using one-way ANOVA and Bonferroni’s test (***indicates *p* ≤ 0.001). The pictures presented in **a**, **b** are representatives of the sample sizes, n = 20 for the whole-mount, and n = 30 for the squash preparations. The same set was used for obtaining the box plots

To have a quantitative measure of the gross synaptic volume occupied by these punctae, we estimated the total number of voxels occupied by the synaptic contacts from the centermost optical slice within a neuromere hemisegment. We then compared this proportional estimate of whole-mount preparations to that of squash preparations. It showed us an increase in the total number of voxels constituting these Bruchpilot stained 3D objects in squash preparations as compared to the whole-mount, with or without size filter (Fig. 5 and Additional file 4: Table S1, Additional file 5: Table S2). This data suggested that more synaptic contacts were captured in squash preparation which could have resulted in the higher proportional estimate.

### Rab4 activation reduces the number of synaptic contacts in the neuropil

Expression of the small GTPase, Rab4, is upregulated in patients with mild cognitive impairment and Alzheimer’s disease in basal cholinergic forebrain and has also been proposed to play a role in axon elongation in *Xenopus* [56, 57]. The Rab4-associated vesicles are shown to be transported by kinesin-2 in *Drosophila* and mammalian cells [52]. Overexpression of the dominant-negative form of Rab4 (S22N, DN) increased the volume of the synaptic region in the ventral nerve cord. Whereas that of the constitutively-active form of Rab4 (Q67L, CA) significantly reduced the volume [52]. The dominant negative form of Rab4 remains in the GDP-bound inactive state and does not allow its binding to the motor protein. It leads to a significant reduction in both recycling and degradation of vesicles. Also, GDP-bound form accumulates in cell bodies present in VNC cortex and reduces the localization of Rab4 to VNC neuropil. In contrast, the constitutively active form of Rab4 would keep it in the GTP-restricted form which will sequester kinesin-2, reducing the availability of the motor for the other cargos [52, 58]. The conjecture was consistent with the observation of reduced neuropil enrichment of choline acetyltransferase (ChAT) in the Rab4^CA^ overexpression background [52]. Both Rab4 and ChAT bind to the C-terminal tail domain of Kinesin-2α [52, 65].

**Fig. 6:**
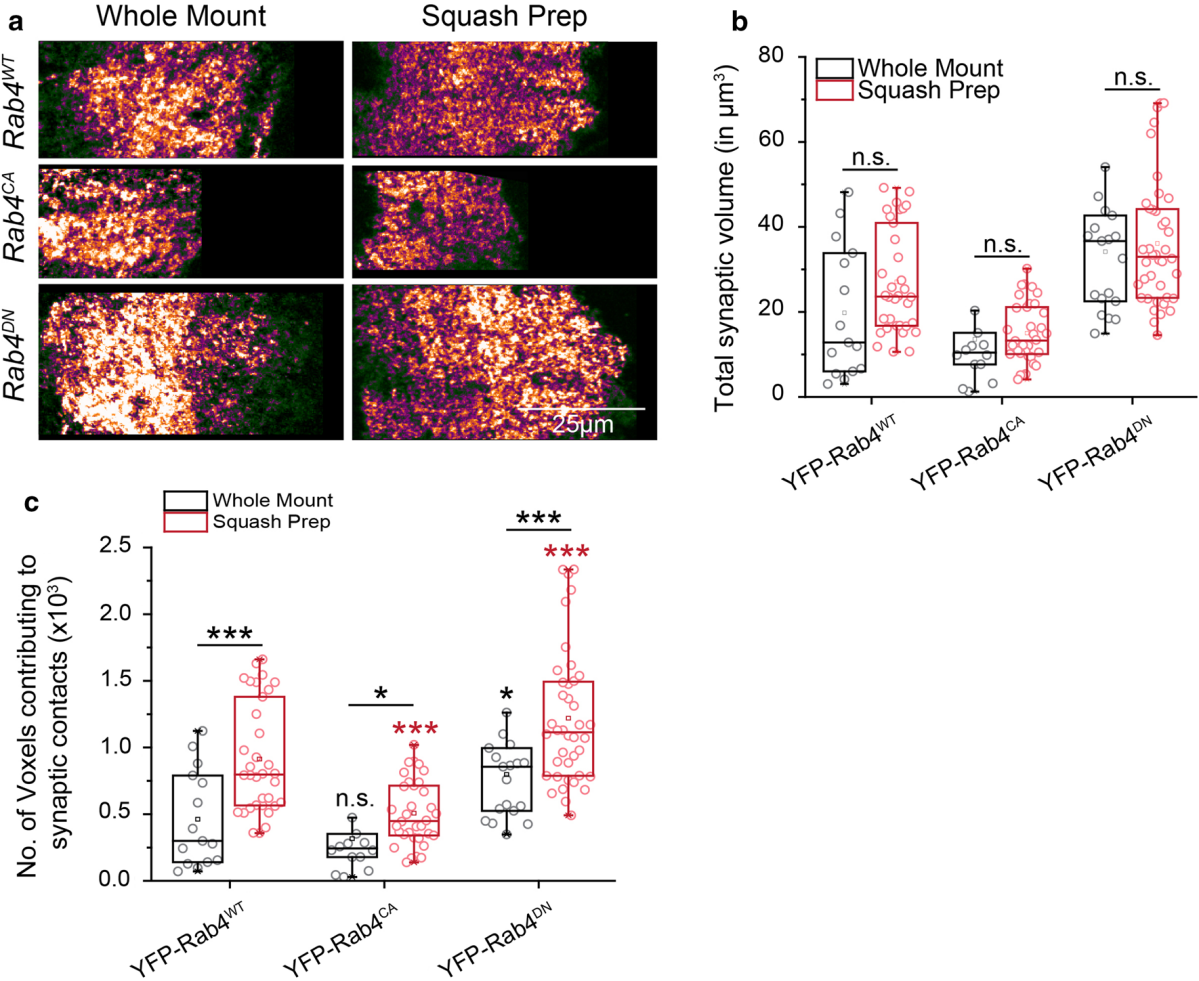
Comparison of synaptic structures between the whole mount and squash preparations in VNCs with different synapse density. **a** Whole-mount and squash preparations of A6 abdominal hemisegments from 78 h AEL VNC, expressing the wild-type Rab4 (*chaGal4, UAS*-*YFPRab4*), constitutively-active Rab4 (*chaGal4, UAS*-*YFPRab4*^*CA*^) and dominant-negative Rab4 (*chaGal4, UAS*-*YFPRab4*^*DN*^) in cholinergic neurons, were stained with the Bruchpilot antibody Magnification ×40 oil objective, N.A. = 1.3; Scale bar: 25 µm for all images in the panel. Images are representatives of the sets used for quantification as described below. **b**, **c** Box plots of the total volume occupied by the synaptic contacts (**b**) and the number of voxels in discrete synaptic contacts (**c**) in a neuromere of the abdominal (A3–A6) hemisegments in the larvae expressing wild-type (WT), constitutively-active (CA), and dominant-negative (DN) forms of Rab4. Comparison between the genotypes was carried out using one way ANOVA and Bonferroni’s test. Pairwise comparison between the whole-mount and squash-prep estimates for each genotype was calculated using two-sample *t* test. Sample sizes for each genotype were n > 14 for the whole-mount estimates, and n ≥ 34 for the squash-prep; the *p* values indicated on each box were obtained with respect to the control values (YFP-Rab4^WT^) from a set of whole-mount (shown in black) and squash-prep samples (shown in red). The underlined *p* values (* < 0.05, *** < 0.001) indicate a pair-wise comparison between the whole-mount and squash-prep from the same genetic background

Therefore, to validate our technique, we overexpressed wild-type (*YFP*-*Rab4*^*WT*^), dominant-negative (*YFP*-*Rab4*^*DN*^) and constitutively-active (*YFP*-*Rab4*^*CA*^) forms of YFP-tagged Rab4 in the cholinergic neurons using *chaGal4*. Estimation of the volume occupied by the synaptic contacts marked by the Bruchpilot antibody in each neuromere (Fig. 6a), both in the whole-mount and in squash preparations, returned similar values (Fig. 6b and Additional file 6: Table S3), indicating that the preparations were morphologically intact. In comparison, estimates of the number of discrete voxels, marked by Bruchpilot in each neuromere, were significantly higher in the squash preparations compared to the values obtained from the whole-mount preparations in all these backgrounds (Fig. 6c and Additional file 6: Table S3). The expression of Rab4^CA^ in cholinergic neurons is expected to reduce gross number of synaptic contacts, whereas the overexpression of Rab4^DN^ is proposed to increase the synaptic contacts [52]. The conclusion was originally derived from the estimates of the average volume occupied by synapses in a neuromere (as shown in Fig. 6b). The counting of Bruchpilot-stained voxels from the whole mount preparations suggested no significant (ns) reduction in the number of synapses in the Rab4^CA^ background and a moderate increase (*p < 0.05) in the Rab4^DN^ background (Fig. 6c). A similar estimate made from the squash preparations suggested highly significant (***p < 0.001) reduction of the synaptic contacts in the Rab4^CA^, and increase in the Rab4^DN^ backgrounds with respect to the wild-type Rab4 control (Fig. 6c). These comparisons helped to highlight that the squash preparation could significantly enhance the sensitivity of the gross synaptic estimation in various backgrounds.

## Discussion

Synapse formation and maturation involve enrichment of various specialized proteins, a variety of lipids and organelles at both the sides [59]. Axonal transport ensures the continuous replenishment of different components at synaptic sites to facilitate efficient neurotransmission [24, 60]. Any defect in axonal transport machinery leads to synapse loss and neurodegenerative disorders [61–63]. *Drosophila* nervous system has been immensely exploited to delineate the molecular mechanism underlying synapse assembly, maintenance and plasticity. Though most of them were studied in NMJs, visual and olfactory system; they were limited to a few types of well defined neurons.

Numerous methods till date have been employed to count synapses in CNS in *Drosophila*. This includes EM and SRM, but there were many pitfalls in using these techniques [36, 41, 45, 46]. Further, EM was not effective in studying molecular mechanism underlying synaptogenesis at a global scale. Moreover, most of the axonal transport deficit phenotypes appeared similar in ultrastructure, so it was difficult to compare the differences among different genetic backgrounds. SRM offered several advantages over EM, but the major drawback was sample thickness and deep tissue imaging [46]. The relatively simple method described above could provide a significant improvements in the data quality obtained from both the Super-resolution and confocal images.

Individual synaptic boutons cannot be resolved by conventional optical microscopy in the CNS due to their compact organization [39, 40, 42]. Therefore, no good assay is established yet for quick assessment of synapse number in whole CNS. Hence, results from neuromuscular studies were extrapolated to CNS, since it was assumed that central nervous system defects would be manifested in NMJs. However, that does not hold true in the case of axonal transport deficits. For example, kinesin-2 mutations show aberrant axonal transport of its cargoes viz. Choline acetyltransferase, Acetylcholinesterase and Rab4 in *Drosophila* sensory neurons [51, 52, 64–66]. However, they do not directly affect synaptic bouton formation in the NMJs [67], compelling us to study the direct impact and correlation of the synapse homeostasis in the CNS neuropil.

The combination of total neuropil volume estimate and voxel count in squash preparation of ventral nerve cord provides a gross sample estimate of synapse density in the neuromere in the larval ventral nerve cord of *Drosophila*. We showed that the estimate also reflects expected changes in the synaptic density in Rab4^CA^ overexpression background. It is possible to extend the method for gross synapse count in the adult brain as well. Also, the method can be applied in diverse genetic backgrounds and for molecular screening of synaptic content. Therefore, it has a potential for wider applications outside the *Drosophila* brain.

## Conclusions

In conclusion, the squash preparation method described above is likely to serve multiple purposes of gross synapse estimation to better resolution of antigenic distribution in the synaptic region using confocal and super-resolution techniques. It is suitable for application in the *Drosophila* larval ganglion and potentially applicable to other brain tissues.

## Additional files

**Additional file 1: Figure S1.** Pre-squash incubation in fixative destroyed tissue morphology. Squash preparation of VNC of *Drosophila* 3rd larval instar. (A and D) GFP-ChAT fluorescence in cholinergic neurons and Bruchpilot staining showing the normal morphology of tissue when tissue was processed without pre-fixation before squash. (B and E) GFP-ChAT fluorescence in cholinergic neurons and Bruchpilot staining when tissue was pre-fixed before squash preparation for 10 minutes, (C and F) pre-fixed 20 minutes. Magnification: 40x oil objective, N.A. =1.3; Scale bars: 25 µm. The images presented here are similar to the observations made in 3-5 such independent VNC preparations.

**Additional file 2: Figure S2.** Milder fixation and permeabilization with 0.3% PT-X yielded a better result. Squash preparation of VNC of *Drosophila* 3rd larval instar. (A, B, C) Bruchpilot staining in pre-fixed squash preparation for various incubations. (D, E, and F) Bruchpilot staining without pre-squash incubation but post-fixation for various incubations. (G, H and I) Bruchpilot staining for various permeabilization treatments. Magnification: 40x oil objective, N.A. =1.3; Scale bars: 25 µm. The images presented here are similar to the observations made in 3-5 such independent VNC preparations.

**Additional file 3: Figure S3.** Method for estimating synaptic contacts from squash preparation using Fiji®. This figure demonstrates the operation of 3D Object Counter plugin of Fiji® software used to grossly assess the number of synaptic junctions in VNC neuromere hemisegment of *Drosophila* third larval instar. A) Abdominal neuromere hemisegment stained with Bruchpilot antibody (pseudo-colored). B) Threshold settings and other parameters for 3D Object Counter plugin, C) Abdominal neuromere hemisegment after applying auto threshold using this plugin, D) Objects map of total 3D objects produced using this plugin in thresholded abdominal neuromere hemisegment, E) Results table displaying the calculated parameters for total number of 3D objects, F) Log window. Magnification: 40x oil objective, N.A. =1.3.

**Additional file 4: Table S1.** The raw data used to produce Figure 5C.

**Additional file 5: Table S2.** The raw data used to produce Figure 5D.

**Additional file 6: Table S3.** The raw data used to produce Figure 6B and C.
