# Supplementary figures and images for: A method for estimating relative changes in the synaptic density in Drosophila central nervous system
Source: BMC Neurosci. 2018 May 16;19:30. doi: 10.1186/s12868-018-0430-3 (PMC5956817; doi:10.1186/s12868-018-0430-3)

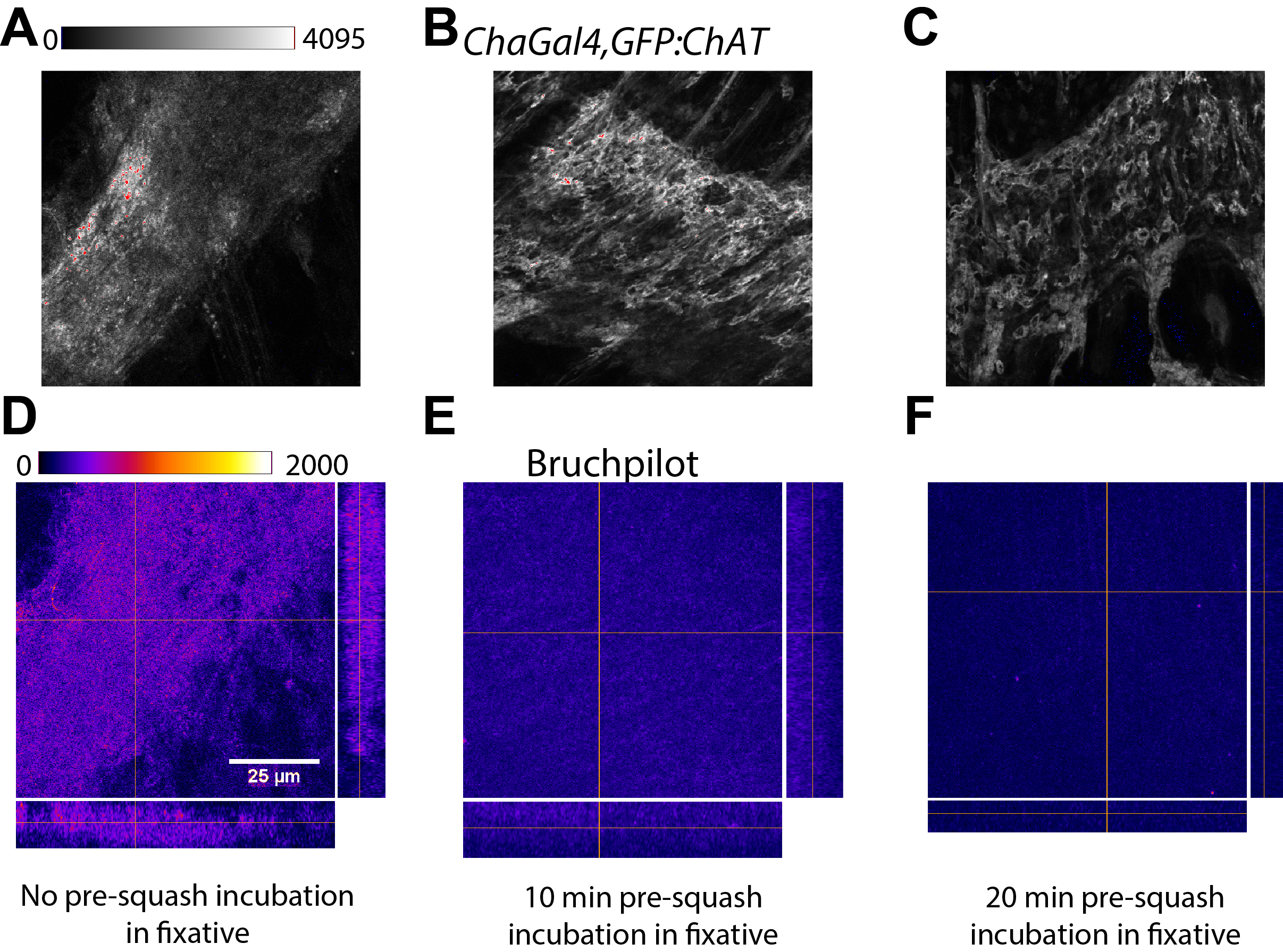

Supplement: Supplementary file 1 — Additional file 1: Figure S1. Pre-squash incubation in fixative destroyed tissue morphology. Squash preparation of VNC of Drosophila 3rd larval instar. (A and D) GFP-ChAT fluorescence in cholinergic neurons and Bruchpilot staining showing the normal morphology of tissue when tissue was processed without pre-fixation before squash. (B and E) GFP-ChAT fluorescence in cholinergic neurons and Bruchpilot staining when tissue was pre-fixed before squash preparation for 10 minutes, (C and F) pre-fixed 20 minutes. Magnification: 40x oil objective, N.A. =1.3; Scale bars: 25 µm. The images presented here are similar to the observations made in 3-5 such independent VNC preparations. [file 12868_2018_430_MOESM1_ESM.jpg]

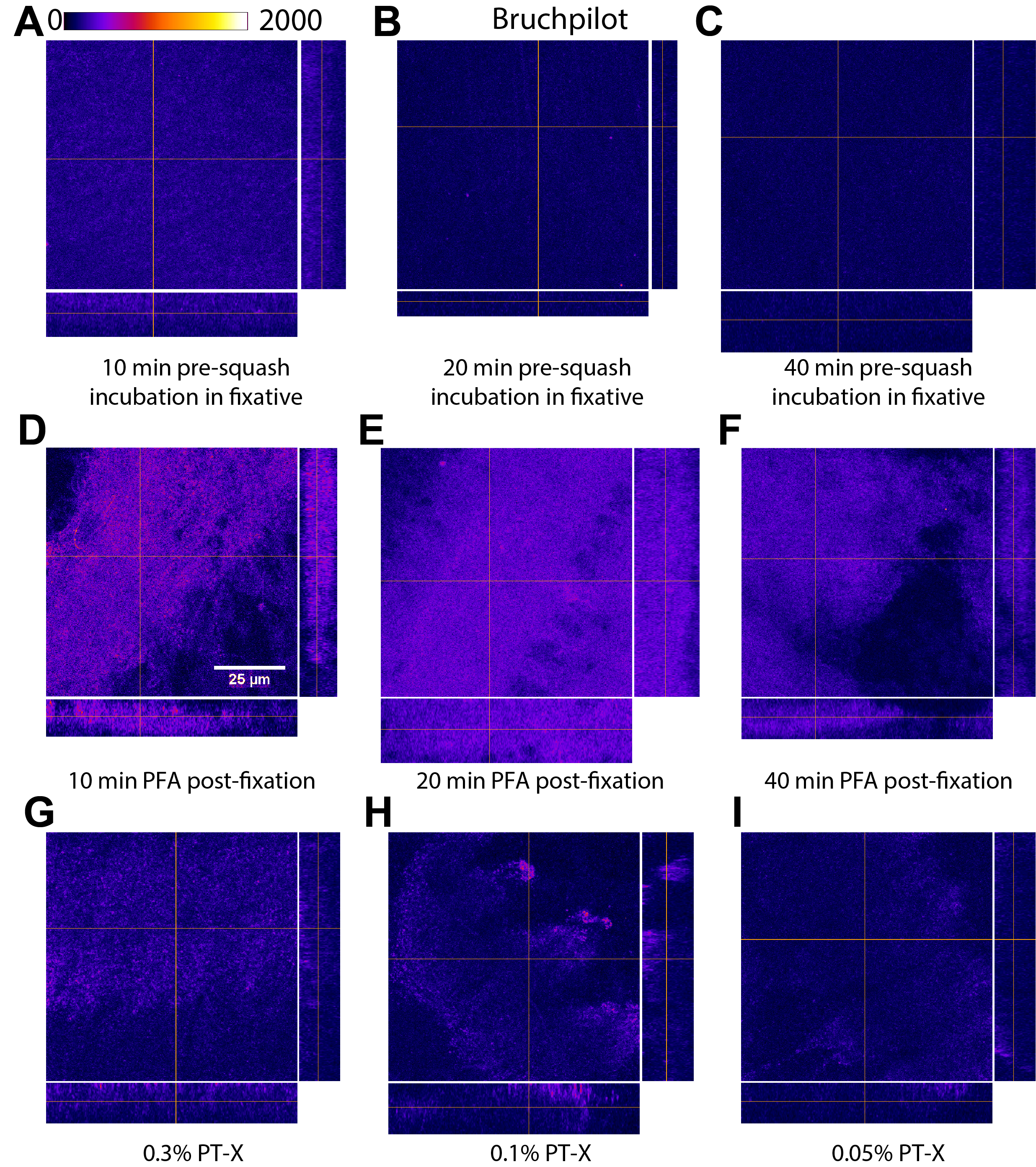

Supplement: Supplementary file 2 — Additional file 2: Figure S2. Milder fixation and permeabilization with 0.3% PT-X yielded a better result. Squash preparation of VNC of Drosophila 3rd larval instar. (A, B, C) Bruchpilot staining in pre-fixed squash preparation for various incubations. (D, E, and F) Bruchpilot staining without pre-squash incubation but post-fixation for various incubations. (G, H and I) Bruchpilot staining for various permeabilization treatments. Magnification: 40x oil objective, N.A. =1.3; Scale bars: 25 µm. The images presented here are similar to the observations made in 3-5 such independent VNC preparations. [file 12868_2018_430_MOESM2_ESM.jpg]

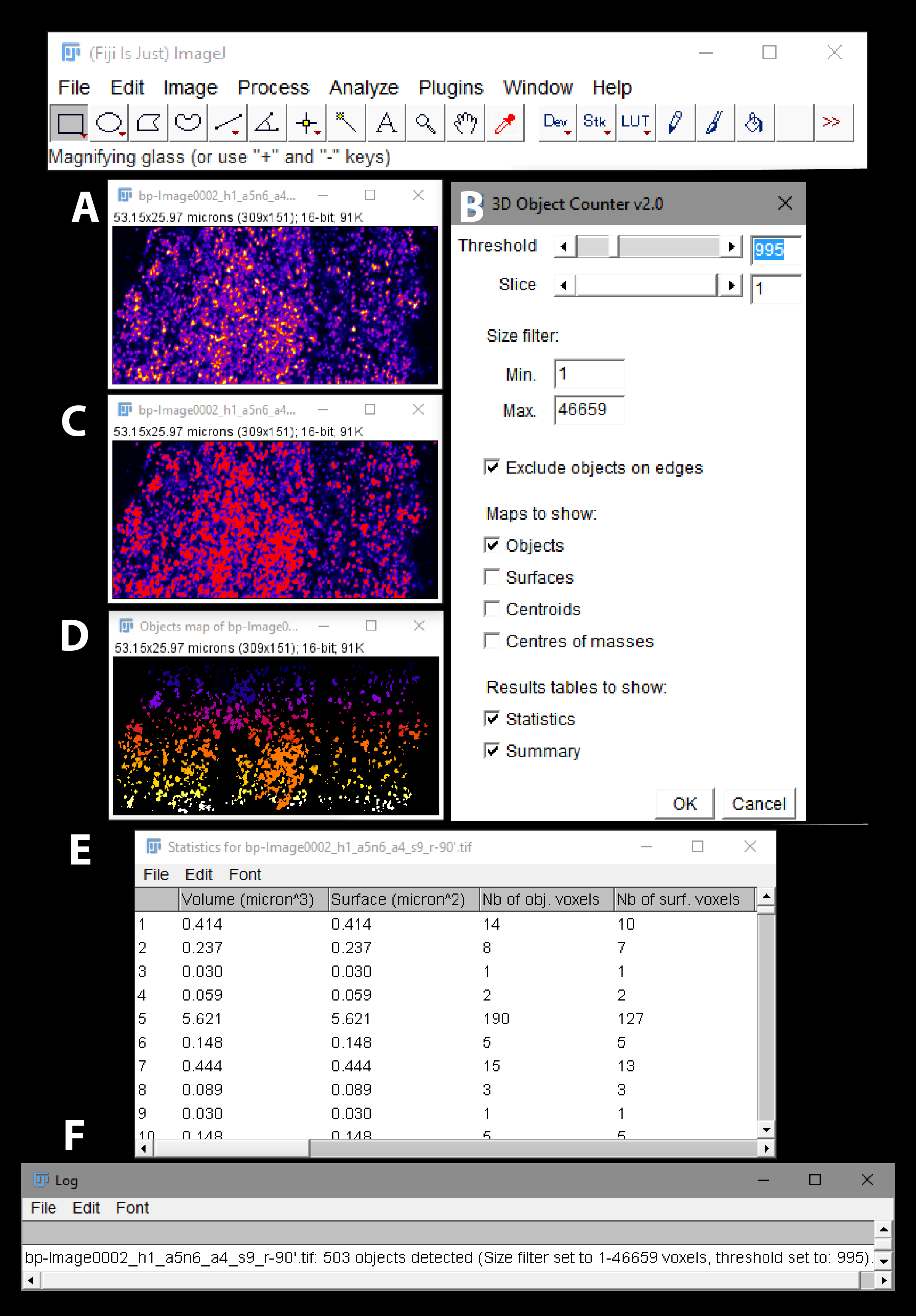

Supplement: Supplementary file 3 — Additional file 3: Figure S3. Method for estimating synaptic contacts from squash preparation using Fiji®. This figure demonstrates the operation of 3D Object Counter plugin of Fiji® software used to grossly assess the number of synaptic junctions in VNC neuromere hemisegment of Drosophila third larval instar. A) Abdominal neuromere hemisegment stained with Bruchpilot antibody (pseudo-colored). B) Threshold settings and other parameters for 3D Object Counter plugin, C) Abdominal neuromere hemisegment after applying auto threshold using this plugin, D) Objects map of total 3D objects produced using this plugin in thresholded abdominal neuromere hemisegment, E) Results table displaying the calculated parameters for total number of 3D objects, F) Log window. Magnification: 40x oil objective, N.A. =1.3. [file 12868_2018_430_MOESM3_ESM.jpg]
